# MCC950, a Selective Inhibitor of NLRP3 Inflammasome, Reduces the Inflammatory Response and Improves Neurological Outcomes in Mice Model of Spinal Cord Injury

**DOI:** 10.3389/fmolb.2020.00037

**Published:** 2020-03-03

**Authors:** Jianhang Jiao, Guanjie Zhao, Yang Wang, Pengfei Ren, Minfei Wu

**Affiliations:** ^1^Department of Orthopedics, The Second Hospital of Jilin University, Changchun, China; ^2^Department of Kidney Medicine, China–Japan Union Hospital of Jilin University, Changchun, China

**Keywords:** spinal cord injury, MCC950, NLRP3, functional recovery, inflammatory response

## Abstract

Spinal cord injury (SCI) is a serious condition that affects bodily function; however, there is no effective therapy in clinical practice. MCC950, a selective NOD-like receptor protein-3 (NLRP3) inflammasome inhibitor, has been reported to alleviate canonical and non-canonical NLRP3 inflammasome activation of the inflammatory response *in vitro* and *in vivo*. However, the effect of MCC950 treatment on neurological post-SCI recovery remains unclear. In this study, we assessed the pharmacological effect of MCC950 on an experimental SCI model *in vivo* and neuronal injury *in vitro*. We found that MCC950 improved the grip strength, hind limb movements, spinal cord edema, and pathological injury in the SCI mice. We demonstrated that it exerted this effect by blocking NLRP3 inflammasome assembly, including NLRP3-ASC and NLRP3-Caspase-1 complexes, as well as the release of pro-inflammatory cytokines TNF-α, IL-1β, and IL-18. Moreover, we found that MCC950 reduced spinal neuron injury and NLRP3 inflammasome activation, which had been induced by oxygen–glucose deprivation (OGD) or lipopolysaccharides (LPS) *in vitro*. In conclusion, our findings indicate that MCC950 alleviates inflammatory response and improves functional recovery in the acute mice model of SCI by blocking NLRP3 inflammasome assembly and alleviating downstream neuroinflammation. Therefore, these findings could prove useful in the development of effective therapeutic strategies for the treatment and prognosis of SCI.

## Introduction

Spinal cord injury (SCI) is a serious condition that affects bodily function. It has been reported to affect over 2.5 million people worldwide with an annual incidence of 40–60 per million populaces (Kumar et al., 2018; Putatunda et al., 2018). In SCI, after the initial mechanical damages, other pathophysiologic mechanisms occur in the spinal cord, including inflammation, oxidative stress, edema, and mitochondrial dysfunction (Quadri et al., 2018), which work toward post-SCI tissue and functional reconstruction (Hausmann, 2003). Unfortunately, there is currently no effective therapeutic strategy in clinical practice for SCI; further, the regulatory molecular mechanisms underlying SCI remain unclear (Donovan and Kirshblum, 2018).

There are various inflammatory complexes present in the central nervous system (CNS) that are preassembled before activation, with the NOD-like receptor protein-3 (NLRP3) appearing to be the most important component (Wallisch et al., 2017; Chen et al., 2019a, b). Previous studies have reported that NLRP3 is mainly expressed in microglia cells; however, recent studies have confirmed that NLRP3 is also expressed in neurons (Chen et al., 2019a, b). NLRP3 inflammasome plays a crucial role in neuroinflammation present in CNS disease (Song et al., 2017; Mortezaee et al., 2018; Wang et al., 2019). NLRP3 inflammasome is assembled by NLRP3, ASC, and Caspase-1 after exogenous infection and endogenous signal stimulation; subsequently, there is Caspase-1 activation and the release of downstream inflammatory factors, such as IL-1β and IL-18 (Yan et al., 2015). Studies have reported NLRP3 inflammasome activation in microglia and neurons after oxygen–glucose deprivation (OGD)/reperfusion *in vitro* and in cerebral ischemia/reperfusion (Gong et al., 2018; Guo et al., 2018). There is post-SCI activation of NLRP3 inflammasome in an animal model involving microglia and spinal cord tissue (Grace et al., 2016; Jiang et al., 2016; Zendedel et al., 2016). Further, previous studies have reported means of reducing neuroinflammation and promoting functional recovery in SCI animals by suppressing NLRP3 inflammasome activity (Qian et al., 2017; Zhu et al., 2017). Jiang et al. (2017) reported that suppression of NLRP3 inflammasome activity to control neuroinflammation using the small-molecule inhibitor BAY 11-7082 or A438079 could attenuate mitochondria damage, alleviate SCI, and improve neuronal function restoration in a mouse model of SCI.

MCC950, a recently developed selective small molecule which is an NLRP3 inflammasome inhibitor, has been reported to reduce canonical and non-canonical activation of NLRP3 inflammasome of inflammatory response *in vivo* and *in vitro* (Corps et al., 2015; Zhang et al., 2017; Hong et al., 2018; Perera et al., 2018; Xu et al., 2018; Chen et al., 2019b). However, the treatment effect of MCC950 on post-SCI neurological recovery remains unclear. In this study, we assessed the pharmacological effects of MCC950 in an experimental SCI-model both *in vivo* and *in vitro*. Further, we examined whether blocking NLRP3 inflammasome assembly and inhibiting its activation through MCC950 treatment could attenuate inflammation and improve neurological post-SCI function. We believe that this study might contribute toward the development of effective therapeutic strategies for the treatment and prognosis of SCI.

## Materials and Methods

### The SCI Model and MCC950 Treatment *in vivo*

We conducted this animal study in accordance with the animal welfare guidelines of the United States National Institutes of Health with supervision and ratification from the Animal Care and Use Committee of the Jilin University. We anesthetized female C57BL/6 mice (25 ± 2 g, 8 weeks) using 3% isoflurane with a rate of 1 L/min. We established the SCI model as previously described (Lin et al., 2016; Jiang et al., 2017); specifically, we made midline skin incisions to expose the T6–T7 spinous processes and conducted laminectomy. Then, SCI was produced by extradural compression of the spinal cord using an aneurysm clip with a closing force of 24 g for 1 min, at the T6 to T7 level. We used mice that underwent laminectomy alone as the sham group. We assessed the SCI conditions at baselines and 1, 2, 3, 7, 14, 21, and 28 days after surgery.

As previously reported (Basso et al., 2006; Yang et al., 2018), we dissolved the NLRP3 inhibitor (MCC950, Selleck, China) in 0.1 M phosphate-buffered saline (PBS) (pH 7.4) with a final concentration of 5 mg/mL and it was administered to animals (10 or 50 mg/kg, i.p.) at 1 and 3 h post-SCI. We treated both the sham and the SCI groups with equal PBS volumes.

### Measurement of Forelimb Grip Strength

We used the Chatillon digital force meter (LTCM-100, Wintop Co., Shanghai, China) to assess the forelimb grip strength. As previously described (Yang et al., 2018), we lifted the mice from their tail and placed them close to the bars of the grasping force gauge to allow them to grasp the bar. Subsequently, we gently pulled the mice back horizontally to pull them off the crossbar with the instrument measuring the grip of the mice’s two front paws. We taped one front paw when assessing the grip of the other front paw. We obtained measurements in triplicates and calculated the average. If a mouse could not grip the crossbar, we recorded the score as 0.

### Neurologic Evaluation

We evaluated hind limb movement during locomotion using the Basso mouse scale (BMS) (Basso et al., 2006), which has a score range of 0 to 9 (from complete paralysis to normal hind limb function). Two researchers who were blinded to the study analysis observed >4 min of open-field exercise at pre-injury and at 1, 2, 3, 7, 14, 21, and 28 days after SCI (*n* = 9 mice/group).

### The Water Content of the Spinal Cord

We assessed the water content in 2 mm spinal cord sections. We immediately weighed the tissues (wet weight) and after dehydration at 100°C for 24 h (dry weight). We calculated the water content of the spinal cord tissue using the following formula: [(wet weight − dry weight)/wet weight] × 100.

### Histological Analysis

We perfused the mice 7 days post-SCI. We carefully obtained the samples, fixed them in 4% paraformaldehyde with subsequent dehydration and paraffin embedding. We stained 4-μ paraffin sections (either proximal or located to the injury site) for histopathological analysis by hematoxylin and eosin (H&E) staining. Next, we observed tissue microscopic alterations in the spinal cord using a microscope. We stained the spinal cord slides using DeadEnd^TM^ Fluorometric terminal deoxynucleotidyl transferase dUTP nick end labeling (TUNEL) System (Promega, Madison, WI, United States) to estimate cell death in the spinal cord tissues. Moreover, we stained cell nuclei using 4′,6-diamidino-2-phenylindole (DAPI). We obtained images using a fluorescence microscope (Olympus, Tokyo, Japan).

### Immunofluorescence Analysis

After fixing with 4% paraformaldehyde, dehydration, and embedding in paraffin, we stained 4-μm thick spinal cord tissue for immunofluorescence (IF) analysis followed by overnight incubation with primary anti-IL-1β (CST, 1:200) and anti-NLRP3 (CST, 1:200) antibodies followed by incubation with secondary antibodies. We stained cell nuclei using DAPI (Sigma-Aldrich) and obtained images using a fluorescence microscope.

### Spinal Neuron Injury Model and MCC950 Administration *in vitro*

We obtained spinal neurons from E16 mouse embryos as described by Pollari et al. (2011) with some modifications. Briefly, after removing the meninges and the dorsal root ganglia, we digested the spinal cord slices using 0.25% trypsin for 15 min at 37°C. We terminated the digestion using horse serum and centrifuged the sample. We resuspended the cells in Dulbecco’s Modified Eagle Media (DMEM) (with 10% fetal bovine serum, 2 mM glutamine), and plated them at a concentration of 2.5 × 10^5^ cells/cm^2^ into poly-D-ornithine-coated (Sigma-Aldrich, St. Louis, MO, United States) plates. The following day, we replaced the medium with Neurobasal Medium [supplemented with 2% B27, 2 mM-glutamine (Invitrogen, Carlsbad, CA, United States) and 10 μM AraC (Sigma-Aldrich)]. After 24 h, we replaced the medium with Neurobasal medium with the aforementioned supplements except for AraC. We changed one-third of the medium every 3 days. We performed subsequent experiments on day 14. To establish a cell model of SCI, we incubated spinal neurons under OGD or lipopolysaccharides (LPS, Sigma-Aldrich).

We employed OGD to simulate ischemic injury *in vitro* (Chen et al., 2011). We exposed the cultured spinal neurons to OGD (glucose-free DMEM, Gibco, United States; 5% CO_2_ and 95% N_2_ for 2 h)/reception (normal medium for 2 h). Moreover, we simulated inflammatory injury via exposure to 10 ng/mL LPS for 4 h and an additional 5 mM adenosine 5′-triphosphate disodium salt hydrate (ATP, Sigma-Aldrich) for 30 min.

As previously described (Mouton-Liger et al., 2018), we immediately suppressed NLRP3 inflammasome activity by adding MCC950 (10 μM, dissolved in PBS) after OGD or LPS exposure. The control group received an equal PBS volume in a similar way.

### Propidium Iodide Staining

We employed PI (Sigma-Aldrich) labeling to detect plasma permeability, which is a hallmark of necrotic cell death. After fixing in 4% paraformaldehyde for 5 min, we washed the cells thrice using PBS followed by staining with 500 nM propidium iodide (PI) for 5 min. We used DAPI (Sigma-Aldrich) to stain nuclei and obtained images using a fluorescence microscope.

### ELISA Analysis

We collected the serum and cell culture medium supernatant. We used a bicinchoninic acid assay kit (Invitrogen, Carlsbad, CA, United States) to determine protein concentration. We conducted ELISA analysis using the TNF-α, IL-1β, and IL-18 ELISA Kit (Anoric-Bio, Tianjin, China) according to the manufacturer’s instructions.

### Lactate Dehydrogenase Release Assay

We assessed neuronal death using an lactate dehydrogenase (LDH) assay kit (Solarbio, Beijing, China). Regarding the spinal neurons, we filtered the supernatant from serum-free media using 0.2-μm syringe filters to use for LDH release detection. We transferred 100 mL of the supernatant to 96-well plates followed by the addition of the reaction mixture and incubation in the dark for 30 min at room temperature. We quantified the LDH concentration by measuring the absorbance at 490 nm using a microplate reader.

### Western Blot Analysis

We used lysis buffer to lyse the spinal cord tissue and primary cultured neurons. After protein quantification, electrophoresis, and Western transfer, we blocked the membranes with 5% bovine serum albumin followed by incubation with the appropriate primary antibodies as follows: NLRP3 (Abcam, 1:1,000), Caspase-1 (Abcam, 1:1,000), Caspase-1 (p20) (AdipoGen, 1:1,000), ASC (Abcam, 1:1,000), LC3 (CST, 1:2,000), and Beclin 1 (CST, 1:1,000), at 4°C overnight. Next, we incubated the membranes with secondary antibodies (Abgent, 1:30,000) followed by the hypersensitivity chemiluminescent substrate (Bio-Rad, United States). We obtained images using a MiVnt image analysis system (Bio-Rad, United States).

### Quantitative Reverse Transcription Polymerase Chain Reaction Analysis

We lysed total RNA using Trizol reagent (Invitrogen, Carlsbad, CA, United States) and performed reverse transcription to cDNA using a HiFi-MMLV cDNA First-Strand Synthesis Kit (CW-Bio, Beijing, China). We conducted quantitative reverse transcription polymerase chain reaction (qRT-PCR) analysis using GoTaq qPCR Master Mix (Promega) and the CFX96TM Real-Time System (Bio-Rad, Hercules, CA, United States). We used *GAPDH* as an internal control. The primer sequences were as follows: NLRP3 F: 5′-GCTAAGAAGGACCAGCCAGAGT-3′, R: 5′-GAACCTGCTTCTCACATGTCGT-3′; GAPDH: F: 5′-AACTTTGGCATTGTGGAAGG-3′ R: 5′-GGATGCAGG GATGATGTTCT-3′.

### Co-immunoprecipitation

We used Pierce IP Lysis Buffer (Invitrogen, Carlsbad, CA, United States) to obtain lysates on ice for 15 min followed by centrifugation to remove the precipitate. After protein quantification, we conducted co-immunoprecipitation (CoIP) using the Dynabeads^TM^ Co-Immunoprecipitation Kit (Invitrogen, Carlsbad, CA, United States) according to the manufacturer’s instructions. Briefly, anti-NLRP3 (Abcam, ab214185) antibody was coupled to Dynabeads with IgG serving as the negative control. Next, we mixed total proteins with antibody-coupled Dynabeads and incubated the mixture overnight at 4°C. Subsequently, we washed the adsorbed Dynabeads with wash buffer and eluted the bounding proteins using elution buffer followed by the addition of equivoluminal 2× Laemmli buffer. Next, we boiled the mixture for 10 min and conducted electrophoresis analysis with 4–20% BeyoGel^TM^ Plus PAGE (Beyotime, Shanghai, China).

### Statistical Analysis

Data are shown as mean ± standard error mean. We conducted Student’s *t*-test or one-way ANOVA followed by Tukey’s multiple comparison test (two-way ANOVA analysis for grip strength assessment) using GraphPad Prism 7.0. We considered *p* < 0.05 as statistically significant.

## Results

### MCC950 Improves the Motor Function in SCI Mice

**FIGURE 1:**
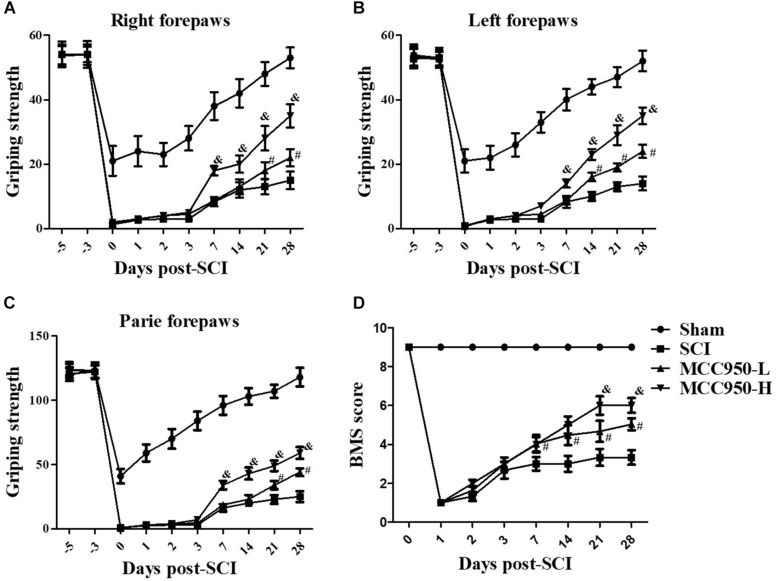
MCC950 ameliorated motor function in SCI mice. We conducted behavioral assessments of mice in the SCI and MCC950 (L: 10, H: 50 mg/kg) groups at 1, 2, 3, 7, 14, 21, and 28 days after surgery. We measured the grip strength of the right forepaw **(A)**, left forepaw **(B)**, and both forepaws **(C)**. **(D)** Daily measurement of the BMS scores. Data are reported as the mean ± SEM, *n* = 10. Two-way ANOVA followed by Tukey’s multiple comparison test, ^#^*p* < 0.05, vs. SCI; ^&^*p* < 0.05, vs. MCC950-L. MCC950 (L: 10 mg/kg, H: 50 mg/kg). BMS, basso mouse scale; SEM, standard error of the mean.

To explore whether MCC950 ameliorated locomotor function in SCI mice, we conducted behavioral assessments using the forelimb grip strength test and BMS score system at 1, 2, 3, 7, 14, 21, and 28 days post-SCI establishment. In the sham group, we found a temporary post-operative decrease in the grip strength, which gradually returned to pre-operative levels after a week. Mice in the SCI group presented serious motor dysfunction of the forelimbs and hind limbs. From 7 to 28 days, we observed a gradual improvement of the grip strength of the left and/or right forelimbs over time; however, serious dyskinesia was still observed (Figures 1A–C). Treatment with GSK872 and Nec-1 did not improve the grip force in the two forelimbs and the accompanying severe dyskinesia within the first 7 days. However, significant improvement of the forepaw function in MCC950-treated (10 or 50 mg/kg) mice began to appear 7 days after injury; moreover, compared to mice in the SCI group, there was a gradual improvement of the grip strength in the MCC950-H-treated mice (50 mg/kg) from 7 to 28 days (*p* < 0.05, Figures 1A–C). Further, the MCC950 protective effect was dose-dependent from 7 days after injury (*p* < 0.05). As shown in Figure 1D, mice in the sham group present slight locomotor post-operative impairment, which quickly returned to normal; however, mice in the SCI group presented severe motor deficits, which recovered after a series of operations. Further, MCC950-treated mice (10 or 50 mg/kg) showed higher scores than the untreated mice. Additionally, we observed a significant difference in the BMS scores between the MCC950-L (10 mg/kg) and MCC950-H groups from 14 days after the operation (*p* < 0.05). These results indicate that MCC950 treatment ameliorates motor function impairment in SCI mice.

### MCC950 Alleviates Spinal Edema and Damage *in vivo*

**FIGURE 2:**
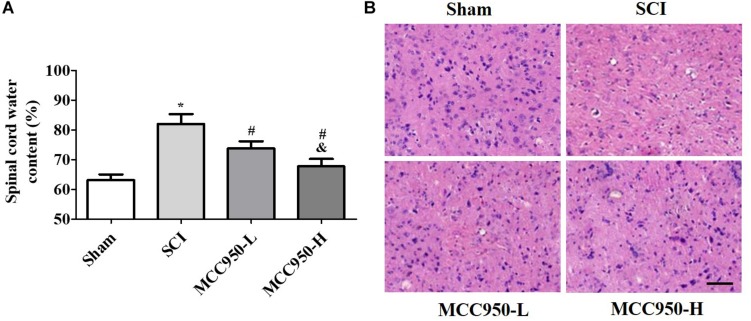
MCC950 alleviated spinal edema *in vivo*. **(A)** Measurement of spinal edema in mice in the MCC950 and SCI groups. **(B)** Representative images of the histological appearance of spinal cord transverse sections after H&E staining. Scale bars: 100 μm. Data are reported as the mean ± SEM, *n* = 9. One-way ANOVA followed by Tukey’s multiple comparison test, *p < 0.05, vs. sham; ^#^*p* < 0.05, vs. SCI; ^&^*p* < 0.05, vs. MCC950-L. MCC950 (L: 10 mg/kg, H: 50 mg/kg). SEM, standard error of the mean.

**FIGURE 3:**
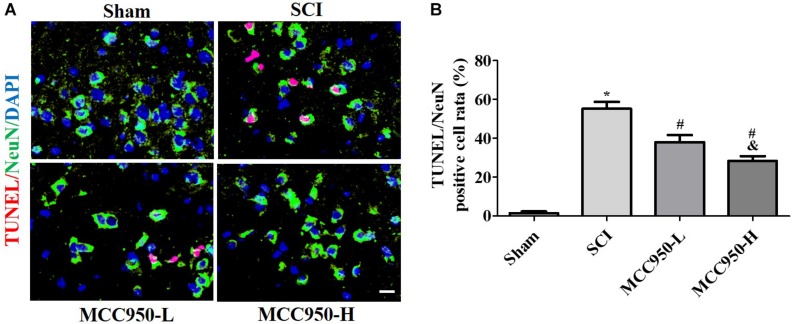
MCC950 reversed spinal cord damage *in vivo*. **(A)** Spinal damage was assessed 7 days post-SCI by TUNEL staining. Nuclei were stained with DAPI and neurons were stained with NeuN. Scale bars: 20 μm. **(B)** Histogram analysis of data on the TUNEL-/NeuN-positive cells. Data are reported as the mean ± SEM, *n* = 9. One-way ANOVA followed by Tukey’s multiple comparison test, *p < 0.05, vs. sham; ^#^*p* < 0.05, vs. SCI; ^&^*p* < 0.05, vs. MCC950-L. MCC950 (L: 10 mg/kg, H: 50 mg/kg). SEM, standard error of the mean.

We assessed the effects of MCC950 treatment on spinal edema *in vivo*. As shown in Figure 2A, there was a prominent post-SCI increase in the wet/dry weight ratio of the spinal tissues after MCC950 treatment (10 or 50 mg/kg) significantly relieving spinal edema in the SCI mice (*p* < 0.05). We used the H&E staining for histopathological analysis (Figure 2B). In the sham group, the cells were arranged in order and remained intact while there was tissue deformation and disorderliness, as well as nucleus shrinkage, in the SCI group. MCC950 treatment (10 or 50 mg/kg, Figure 2B) alleviated the spinal tissue damage. We also measured spinal cord damage using TUNEL staining (Figure 3A). As shown in Figure 3A, a large number of spinal neurons stained with red fluorescence, indicating severe nerve damage at 7-day post-SCI. MCC950 treatment significantly reduced TUNEL-/NeuN-positive cells (Figure 3B) with high-dose MCC950 (50 mg/kg) appearing to have better protective effect than low-dose MCC950 (*p* < 0.05).

### MCC950 Blocks NLRP3 Inflammasome Activity and Post-SCI Inflammatory Reaction *in vivo*

**FIGURE 4:**
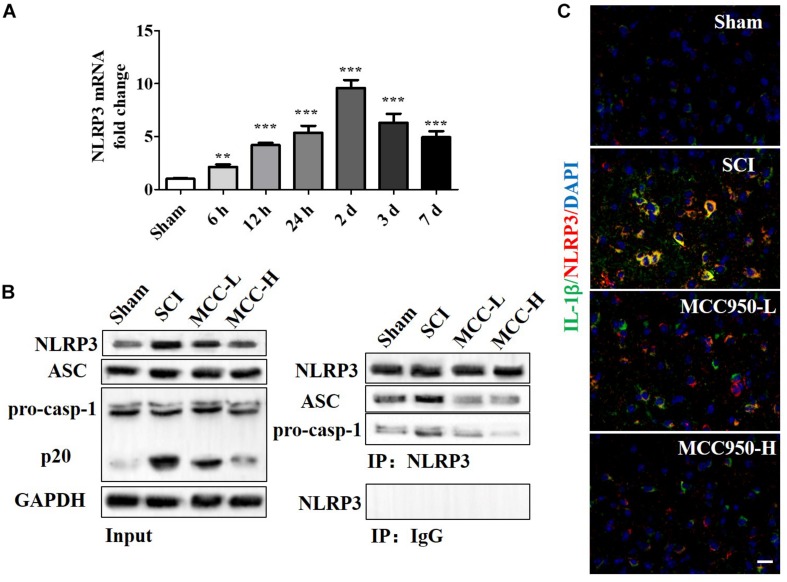
MCC950 blocks NLRP3 inflammasome activation post-SCI *in vivo*. **(A)** We assessed NLRP3 mRNA levels at 7 days post-SCI. **(B)** We conducted a CoIP assay to investigate NLRP3 inflammasome assembly, including the mutual combination of NLRP3 and ASC and of NLRP3 and pro-Caspase-1 at 7 days post-SCI. Left: WB analysis of NLRP3, ASC, and Caspase-1 (p20) levels in lysates/input. Right: We conducted the CoIP using an anti-NLRP3 antibody and subjected the CoIP products to WB analysis for NLRP3, ASC, and pro-Caspase-1 levels. **(C)** IF analysis of IL-1β and NLRP3 levels in the SCI and MCC950 groups. Scale bars: 20 μm. Data are reported as the mean ± SEM, *n* = 8. One-way ANOVA, **p < 0.01, ***p < 0.001, vs. sham. MCC950 (L: 10 mg/kg, H: 50 mg/kg). CoIP, co-immunoprecipitation; WB, western blot; IF, immunofluorescence; SEM, standard error of the mean.

We measured the mRNA and protein NLRP3 expression levels at 7-day post-SCI (Figures 4A,B). Compared to the sham group, there was a significant increase in the NLRP3 mRNA levels at 7-day post-SCI (Figure 4A, *p* < 0.05). The CoIP assay confirmed that SCI induced NLRP3 inflammasome assembly, including the mutual combination of NLRP3 and ASC and of NLRP3 and pro-Caspase-1 (Figure 4B). Compared to the SCI group, MCC950 treatment suppressed NLRP3 expression and NLRP3 inflammasome assembly; further, it slightly suppressed pro-Caspase-1 and ASC expression and significantly reduced the levels of the p-20 fragment of Caspase-1 (Figure 4B). Figure 4C presents the IF analysis of the IL-1β and NLRP3 levels. There was a post-SCI increase in the IL-1β/NLRP3 levels with MCC950 treatment and reduced NLRP3 expression and IL-1β levels.

**FIGURE 5:**
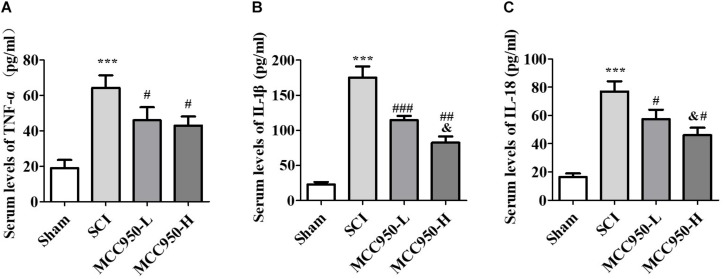
Serum levels of TNF-α **(A)**, IL-1β **(B)**, and IL-18 **(C)** at 7 days post-SCI. There was a notable decrease in the TNF-α, IL-1β, and IL-18 levels in the MCC950 (L and H) groups compared to the SCI group. Data were reported as the mean ± SEM, *n* = 9. One-way ANOVA followed by Tukey’s multiple comparison test, ***p < 0.001, vs. sham. ^#^*p* < 0.05, ^##^*p* < 0.01, ^###^*p* < 0.01, vs. SCI. ^&^*p* < 0.05, vs. MCC950-L. MCC950 (L: 10 mg/kg, H: 50 mg/kg). SEM: standard error of the mean.

We used ELISA to measure the serum levels of TNF-α, IL-1β, and IL-18 at 7-day post-SCI (Figure 5). Compared to the sham group, we found a significant increase in the serum TNF-α, IL-1β, and IL-18 levels at 7-day post-SCI. Compared to the SCI group, MCC950 treatment significantly reduced the pro-inflammatory cytokines TNF-α (Figure 5A), IL-1β (Figure 5B), and IL-18 (Figure 5C). Additionally, the MCC950-induced reduction in IL-1β and IL-18 levels was dose-dependent (*p* < 0.05).

### MCC950 Mitigates Neuron Injury and NLRP3 Inflammasome Activation *in vitro*

**FIGURE 6:**
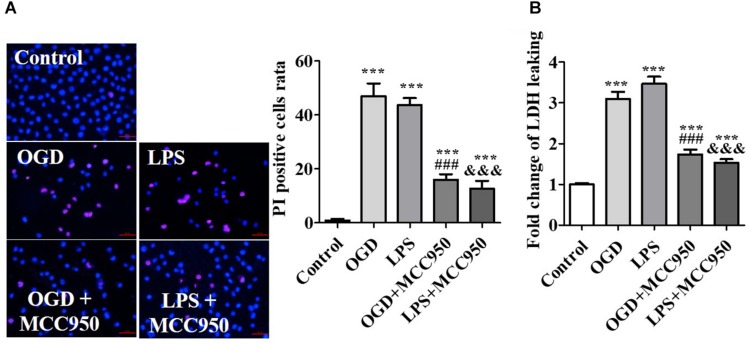
MCC950 prevents spinal neuron injury. **(A)** Representative images of PI staining examined after treatment of OGD- or LPS-exposed spinal neurons with MCC950 (50 μM). We statistically analyzed data of the PI-positive cells. **(B)** Moreover, we conducted the LDH assay to assess neuronal death. Data are reported as the mean ± SEM, *n* = 5. ***p < 0.001, vs. control. ^###^*p* < 0.001, vs. OGD. ^&&&^*p* < 0.001, vs. LPS. OGD, oxygen–glucose deprivation; LPS, lipopolysaccharides; PI, propidium iodide; SEM, standard error of the mean.

Mimicking the pathological conditions of SCI *in vitro*, spinal cord neurons were subjected to OGD or LPS. OGD and LPS have been widely used in the study of nerve injury of SCI *in vitro* (Li et al., 2019; Zhang et al., 2019). On the 14th day of the *in vitro* culture, we used the cells for subsequent experiments. To demonstrate the inhibitory effect of MCC950 on neuron injury, we conducted PI staining analysis to assess the effect of MCC950 treatment on OGD- or LPS-exposed spinal neurons. We detected the number of PI-positive cells after OGD- or LPS-exposure as PI is impermeable to intact cell membranes and labels damaged cells. We found very few PI-positive cells in the control group. Conversely, there was an increase in PI-positive cells after OGD- or LPS-exposure, which were remarkably reduced after MCC950 treatment (*p* < 0.001; Figure 6A). Further, we performed the LDH assay to assess neuronal death. We found that OGD and LPS significantly increased LDH release and that MCC950 treatment significantly alleviated LDH release (*p* < 0.001; Figure 6B).

**FIGURE 7:**
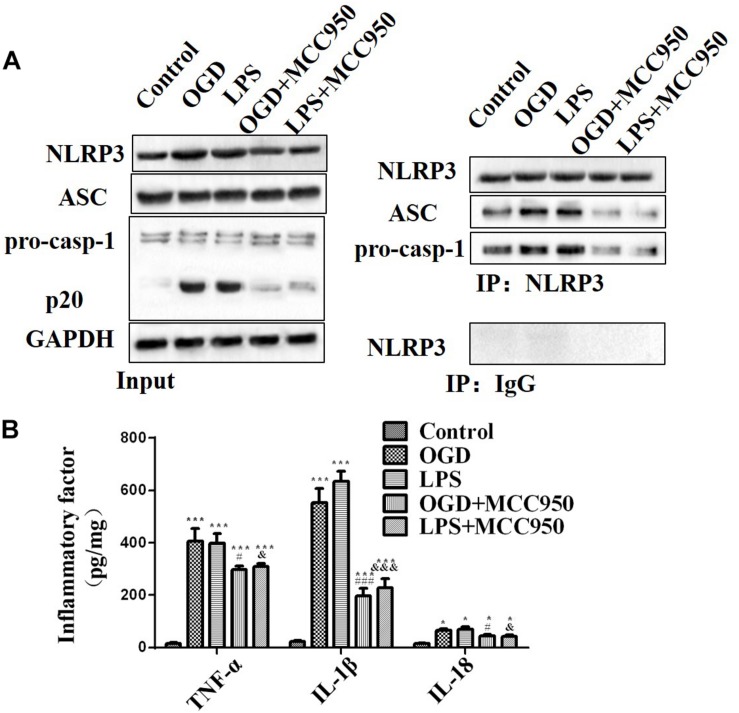
MCC950 inhibits NLRP3 inflammasome activity and pro-inflammatory cytokine release. **(A)** We conducted a CoIP analysis to assess MCC950 (50 μM)-induced inhibition of NLPR3 inflammasome activation in OGD- or LPS-exposed spinal neurons. Left: WB analysis of NLRP3, ASC, and Caspase-1 (p20) levels in lysates/input. Right: We conducted CoIP analysis using an anti-NLRP3 antibody and subjected the CoIP products to WB analysis of NLRP3, ASC, and pro-Caspase-1 levels. **(B)** The levels of TNF-α, IL-1β, and IL-18 release. Data were reported as the mean ± SEM, *n* = 5. One-way ANOVA, *p < 0.05, ***p < 0.001, vs. control; ^#^*p* < 0.05, ^###^*p* < 0.001, vs. OGD; ^&^*p* < 0.05, ^&&&^*p* < 0.001, vs. LPS. OGD, oxygen–glucose deprivation; LPS, lipopolysaccharides; CoIP, co-immunoprecipitation; WB, Western blot; SEM, standard error of the mean.

We examined whether MCC950 treatment altered the NLRP3 inflammasome activation pathway in spinal neurons after OGD or LPS exposure. We analyzed the NLRP3 inflammasome assembly and pro-inflammatory cytokine release in mice treated with MCC950 and untreated. As shown in Figure 7A, OGD and LPS exposure significantly induced NLRP3 inflammasome activity with MCC950 treatment dramatically reducing the assembly of NLRP3-ASC and NLRP3-pro-Caspase-1 complexes compared to non-treatment after OGD or LPS exposure. Moreover, MCC950 decreased TNF-α, IL-1β, and IL-18 (Figure 7B, *p* < 0.001) release, which had been notably increased by OGD or LPS exposure.

## Discussion

In this work, we demonstrated that it exerted this effect by blocking NLRP3 inflammasome assembly, including NLRP3-ASC and NLRP3-Caspase-1 complexes, as well as the release of pro-inflammatory cytokines TNF-α, IL-1β, and IL-18. Moreover, we found that MCC950 reduced spinal neuron injury and NLRP3 inflammasome activation, which had been induced by OGD or LPS *in vitro*. In conclusion, our findings indicate that MCC950 alleviates inflammatory response and improves functional recovery in the acute mice model of SCI by blocking NLRP3 inflammasome assembly and alleviating downstream neuroinflammation.

Previous studies have reported that SCI mainly occurs after a violent collision or trauma and can trigger adverse complications, including movement disorder and paralysis (Kudo et al., 2006; Lv et al., 2018). Inflammasome-mediated signals are crucial in immune responses, as well as in metabolism and microbial infections, and involve various pressure and damage signals that immediately activate Caspase-1 with subsequently increased secretion of potent pro-inflammatory cytokines and pyroptosis (Strowig et al., 2012). There has been increasing attention on the molecular mechanisms of NLRP3 in a number of diseases. Recent studies have reported significant protective effects resulting from suppression of NLRP3 inflammasome activation in liver fibrosis, systemic sclerosis, systemic lupus erythematosus, systemic sclerosis, diabetes, inflammasome-related eye disease, inflammatory bowel disease, rheumatoid arthritis, and CNS diseases (Zhou et al., 2016; Alegre et al., 2017; Rovira-Llopis et al., 2018; Shen et al., 2018; Yerramothu et al., 2018; Chen et al., 2019a). Meng et al. (2019) demonstrated that PPAR-γ activation exerts an anti-inflammatory effect by suppressing NLRP3 inflammasome activation in spinal cord-derived neurons. Further, NLRP3 inflammasome activation has been reported in microglia and neurons after OGD/reperfusion *in vitro* and during cerebral ischemia/reperfusion (Gong et al., 2018; Guo et al., 2018). Therefore, we speculated that NLRP3 inflammasome might be a promising therapeutic target for SCI. However, the role of MCC950 as an effective inhibitor for SCI treatment has been poorly studied. Our findings indicated that MCC950, which is a selective NLRP3 inflammasome inhibitor, blocks NLRP3 inflammasome assembly and mitigates inflammation and neurological dysfunction in SCI mice.

Spinal cord injury is a pathological event that is inflammation-regulated and causes axonal injury, neuron loss, and demyelination during the secondary cascade of injury (de Rivero Vaccari et al., 2008; Mortezaee et al., 2018). In SCI, violent collision or trauma directly causes primary injury limited to the vertebral fracture region in the spinal cord with subsequent acute hemorrhage, necrotic cell death, and neuron loss (Lozano et al., 2015; Lin et al., 2016). Secondary injury leads to diffuse and long-term injury that destroys gliocytes and neurons and triggers neuron death and neurodegeneration followed by an apparent spread of the injury to higher spinal cord segments (Lin et al., 2016; Mortezaee et al., 2018).

Studies have reported post-SCI immoderate NLRP3 expression in neurons and gliocytes with neurons being the main cell type that expresses NLRP3 inflammasome (Qian et al., 2017; Mortezaee et al., 2018). A time-independent significant increase in NLRP3 inflammasome has been reported at 72 h after SCI in rat and mouse models (Grace et al., 2016; Jiang et al., 2017). Jiang et al. (2017) reported that NLRP3 mRNA and protein expression levels were the highest 3 days post-SCI in mice; however, there was no significant change in NLRP3 protein levels within 24 h. Contrastingly, our study found a gradual increase in NLRP3 mRNA expression within the initial 24 h post-SCI, which declined at 48 h and increased again at 72 h. This might be attributed to the direct death of neurons or glial cells caused by mechanical injury at the initial stage, which causes NLRP3 activation with the body’s protective mechanism being immediately activated to reduce the NLRP3 upregulation. This phenomenon is consistent with the trend of NLRP3 levels in the cerebrospinal fluid of patients with clinical traumatic brain injury (TBI) (Wallisch et al., 2017).

Pharmacologic inhibitors of NLRP3 inflammasome are currently at an early development stage. MCC950, which is the most specific and well-characterized NLRP3 inhibitor, has been validated in multiple NLRP3-related inflammatory diseases (Perera et al., 2018; Chen et al., 2019b). Multiple studies have assessed the effect of MCC950 in the CNS. Ren et al. (2018) reported that MCC950 reduces IL-1β production and attenuates neurological dysfunctions after intracerebral hemorrhage (ICH) induced by the injection of either autologous blood or collagenase. The protective effect of MCC950 has been related to the reduction of brain leukocyte infiltration and microglial IL-6 production in ICH mice, as well as improvement of blood–brain barrier (BBB) integrity and decreased neuron death (Ren et al., 2018). Moreover, Ye et al. (2017) reported that BV2 cell apoptosis and apoptotic cell protein expression were significantly attenuated after MCC950 treatment in thrombin-induced brain injury. Qi et al. (2018) reported that MCC950 treatment reversibly reversed the inhibition of long-term potentiation and improved synaptic plasticity deficits in an Alzheimer’s disease rat model. Ismael et al. (2018) reported that MCC950 alleviates pro-inflammatory sand pro-apoptotic signals in the acute phase of TBI. Xu et al. (2018) confirmed that MCC950 reduces inflammation and improves long-term neurological outcomes in a murine model of TBI by reducing microglial activation, leukocyte recruitment, and pro-inflammatory cytokine production, as well as BBB integrity preservation and cell death attenuation. In our study, we observed that MCC950 significantly improved and accelerated recovery of forepaw function and neurological function. We observed a significant dose-dependent reduction of hemorrhage, edema, inflammatory cells, vacuoles, and neuron death in the spinal cord of SCI mice after MCC950 treatment. Further, our findings demonstrated excellent protective features of MCC950 that involved repressing NLRP3 inflammasome assembly, controlling Caspase-1 activation (Caspase-1 p20), and inhibiting the release of TNF-α, IL-1β, and IL-18. In neuronal cells, Zhang et al. (2019) imitate SCI by using OGD model, they verify that overexpression of miR-21 suppresses caspase-3 activity and apoptosis via targeting PDCD4. To evaluate the ability of Melatonin to enhance autophagy and inhibit apoptosis via activation of the PI3K/AKT/mTOR signaling pathway, Li et al. (2019) demonstrate that Melatonin reduces LPS-induced autophagy and apoptosis in primary spinal cord neurons by inhibition PI3K/AKT/mTOR pathway. We showed MCC950 also alleviated OGD- and LPS-induced primary spinal cord neuronal damage and inflammation via repressing NLRP3 inflammasome active *in vitro*. However, Xu et al. (2018) reported that MCC950 reverses the counts of microglia (CD11b + CD45int), macrophages (CD11b + CD45hiLy6G^–^), neutrophils (CD11bCD45hi + Ly6G^+^), CD4 + T cells (CD45hiCD3 + CD4^+^), and CD8 + T cells (CD45hiCD3 + CD8^+^) after TBI with the effect on microglia being the most significant. Future studies should investigate whether MCC950 further regulates post-SCI inflammatory signals by affecting certain glial cell types.

## Conclusion

In conclusion, our findings indicate that MCC950 can reduce the inflammatory response and improve functional recovery in the acute mice model of SCI by blocking NLRP3 inflammasome assembly and alleviating downstream neuroinflammation. There is a need for future studies to completely elucidate the relationship between MCC950 and NLRP3 inflammasome after SCI, the role of MCC950 in glia activation and transition and synaptic plasticity after SCI, and its clinical feasibility including safety issues regarding MCC950-associated therapies in preclinical settings. Our findings might contribute toward the development of effective therapeutic strategies for the treatment and prognosis of SCI.

## Data Availability Statement

The datasets generated for this study are available on request to the corresponding author.

## Ethics Statement

This study was carried out in accordance with the principles of the Basel Declaration and recommendations of the animal welfare guidelines of the United States National Institutes of Health, as well as the Animal Care and Use Committee of Jilin University. The protocol was reviewed and approved by the Animal Care and Use Committee of Jilin University.

## Author Contributions

JJ and MW designed the work. GZ developed the methodology. YW and PR performed the experiments. JJ and YW performed the data analysis and created the figures. JJ wrote the first draft. MW reviewed and revised the manuscript, and supervised the study. All the authors have read and approved the manuscript.

## Conflict of Interest

The authors declare that the research was conducted in the absence of any commercial or financial relationships that could be construed as a potential conflict of interest.
